# Aspirin hypersensitivity in Iranian patients with chronic rhinosinusitis and nasal polyposis: prevalence and comorbid factors

**DOI:** 10.1186/2045-7022-4-S3-P22

**Published:** 2014-07-18

**Authors:** Hossein Esmaeilzadeh, Mohammad Nabavi, Saba Arshi, Mohammad Hassan Bemanian, Ahmad Bahrami, Morteza Fallahpour, Negar Mortazavi, Nima Rezaei

**Affiliations:** 1 Tehran University of Medical Science, Iran; 2 Iran University of Medical Science, Allergy and Clinical Immunology, Iran; 3 Tehran University of Medical Science, Allergy and Clinical Immunology, Iran; 4 Shahid Beheshti Medical University, Clinical Pharmecy Department, Iran; 5 Tehran University of Medical Science, Molecular Immunology Research Center, Iran

## Background

Aspirin (ASA) hypersensitivity is frequent in patients with chronic rhinosinusitis and nasal polyps (CRSwNP), which is called aspirin exacerbated respiratory disease (AERD). However, there are few studies on evaluating the prevalence of ASA hypersensitivity in patients with nasal polyps (NPs), using the oral aspirin challenge test. This study was designed to determine the frequency of ASA hypersensitivity and factors associated with ASA hypersensitivity in patients with CRSwNP.

## Methods

Eighty Iranian patients (43 women and 37 men) with CRSwNP were enrolled in this study. Extension of NPs was evaluated by endoscopic examination. A single day, oral aspirin challenge (OAC) was used to detect ASA hypersensitivity. Atopic evaluation was performed, using skin prick test, nasal smear, blood eosinophil count, and serum total IgE.

## Results

OAC was performed in all patients (mean age: 38.9± 10.7 years). OAC resulted positive in 39 patients (48.8%); among them 14 (35.8%) had history of ASA hypersensitivity, while 2 patients (12.5%) with positive history were negative through OAC. Concomitant asthma, pervious polyp surgery, high polyp score and ASA hypersensitivity history were all associated with positive OAC (p<0.05). Presence of AERD was not associated with age, stage of asthma, blood eosinophilia, nasal smear eosinophilia and atopy.

## Conclusion

ASA hypersensitivity is quite common in patients with CRSwNP. Patients at risk for AERD require evaluation for the presence of ASA hypersensitivity with ASA provocation challenge test to confirm the diagnosis.

